# Genomic loss of *MLH1/PMS2* loci defines a mismatch repair deficient subgroup in monomorphic epitheliotropic intestinal T-cell lymphoma

**DOI:** 10.1038/s41408-026-01562-3

**Published:** 2026-07-03

**Authors:** Luis Veloza, Anja Fischer, Vimel Rattina, David Vallois, Rita Sarkis, Karine Lefort, Bettina Bisig, Doriane Cavalieri, Olivier Tournilhac, Philippe Gaulard, Reiner Siebert, Laurence de Leval, Edoardo Missiaglia

**Affiliations:** 1 Institute of Pathology, Department of Laboratory Medicine and Pathology, Lausanne University Hospital and Lausanne University, Lausanne, Switzerland; 2 Institute of Human Genetics, Ulm University and Ulm University Medical Center, Ulm, Germany; 3 Department of Hematology, Lille University Hospital, Lille, France; 4 Adult Clinical Hematology, CHU Clermont-Ferrand, Clermont Auvergne University, Clermont-Ferrand, France; 5 Paris-Est Créteil University, Faculty of Medicine and Health, Campus Henri Mondor; Department of Pathology, Henri Mondor University Hospital, Créteil, France; 6 German Center for Child and Adolescent Health (DZKJ), Partner site Ulm, Ulm, Germany

**Keywords:** Cancer genomics, Translational research

## Abstract

Primary intestinal T-cell lymphomas (ITCLs), comprising enteropathy-associated T-cell lymphoma (EATL) and monomorphic epitheliotropic intestinal T-cell lymphoma (MEITL), are rare aggressive tumors. The role of DNA mismatch repair (MMR) deficiency (dMMR) and microsatellite instability (MSI) in the development of ITCLs remains largely unexplored. Here, we investigated the incidence, molecular mechanisms and clinical relevance of dMMR/MSI in 86 ITCLs (30 EATLs, 56 MEITLs) using whole-exome sequencing, PCR-based MSI testing, DNA methylation profiling, and immunohistochemistry for MLH1, MSH2, MSH6 and PMS2. MMR deficiency was detected in 3 of 53 MEITLs (6%) but in none of the EATLs. dMMR MEITLs showed the highest tumor mutational burden (8.3–17.1 mutations/Mb), compared to median TMBs of 1.9 in MEITL and 2.4 in EATL. The complete loss of MLH1/PMS2 expression in two MSI-high tumors and isolated PMS2 loss in the third case were all associated with biallelic deletions of the affected loci. Notably, *MLH1* deletions significantly co‑occurred with *SETD2* deletions (*p* = 0.001), the latter representing a major driver of MEITL tumorigenesis. dMMR MEITLs lacked distinctive clinicopathologic features. These findings indicate that dMMR/MSI occurs in a subset of MEITLs probably during tumor progression, rather than being an initiating driver event, and provide a biological rationale to explore the efficacy of immune checkpoint inhibitors in some of this unfavorable subtype of ITCL.

## Introduction

Genomic instability is a hallmark of cancer cells, and microsatellite instability (MSI), resulting from defects in the DNA mismatch repair (MMR) system, plays a significant role in driving this process. The MMR system corrects single base mismatches and small insertion-deletion loops (IDLs) of unpaired bases generated during DNA replication and recombination. Deficiency of MMR (dMMR) proteins favors the accumulation of mutations, especially in microsatellite repeat regions, leading to a higher tumor mutational burden (TMB) and increased neoantigen expression, which may induce antitumor T-cell activation [1].

Deficient MMR occurs in various types of cancer, with the highest incidence in endometrial (20-30%), colorectal (10-15%) and gastric (10%) carcinomas, where it can arise from somatic or constitutional alterations in the MMR genes [2]. While 80% of dMMR colorectal carcinomas are due to epigenetic silencing of *MLH1* promoter, the remaining cases are caused by germline pathogenic variants in one of the MMR genes (*MSH2, MLH1, MSH6* and *PMS2*), as observed in Lynch Syndrome, accounting for 2% to 4% of all colorectal carcinomas and approximately 2.5% of endometrial carcinomas [3, 4]. Moreover, MSI status has important predictive and prognostic implications in some solid tumors, where high microsatellite instability (MSI-H) and dMMR are associated with a better response to immune checkpoint inhibitors (ICIs) when compared to those with stable (MSS) or low (MSI-L) microsatellite instability status [5]. Information about MSI status in hematological malignancies is sketchier, with contrasting ranges of frequencies, undefined clinical implications, and unclear underlying molecular mechanisms [6–19]. Notably, only one recent study has suggested a predictive impact of MSI status in diffuse large B-cell lymphoma (DLBCL) [12].

Our group has a longstanding interest in primary intestinal T-cell lymphomas (ITCLs), which comprise two main entities: enteropathy-associated T-cell lymphoma (EATL) and monomorphic epitheliotropic intestinal T-cell lymphoma (MEITL)[20, 21]. By exploring their genetic and molecular profiles, our works demonstrated partially overlapping features with diverging attributes. We showed that MEITLs are characterized by *SETD2* loss-of-function alterations, *H3-3A/B* mutations, and *JAK3*/*STAT5B* pathway alterations, along with promoter CpG hypomethylation [20, 21]. In contrast, EATLs harbor frequent *TET2*, *ARID1A*, and *KMT2D* mutations, *JAK1*/*STAT3* alterations, and HLA class I disruptions, with an immunosuppressive microenvironment marked by macrophage enrichment and enhanced inflammatory gene expression [21]. Clinically, both diseases affect predominantly older adults who often present with intestinal perforation and/or obstruction [20, 21]. EATLs have historically had a poor prognosis [22] especially in the context of refractory sprue, which complicates their management, but prospective studies show that survival can be improved with a strategy of polychemotherapy and autologous transplantation [23, 24] with even better results in a recent study incorporating anti-CD30 antibody-drug conjugate [25]. In MEITL, which is CD30 negative and with a median overall survival of less than 10 months, no study suggests that the poor outcome can be improved [20]. Thus, the development of new therapeutic approaches is warranted, and there is a critical unmet medical need in MEITL.

To date, the frequency of dMMR/MSI in ITCLs is largely unknown. In 2003, Baumgärtner et al. studied genomic aberrations and genomic instability in a series of 26 EATL (before the new established classification distinguishing EATL and MEITL) using 47 microsatellite repeat markers, reporting instability in 69% of cases (all MSI-L) [15]. The present study aims to explore the role of dMMR/MSI in a large and well-characterized series of ITCLs, to assess the underlying molecular mechanisms and correlation with pathological and clinical features.

## Material and Methods

### Patients and samples

A total of 86 cases (30 EATLs and 56 MEITLs) diagnosed between 2000 and 2022 were collected from several institutions in Switzerland, France, Belgium and Germany and through the T-cell lymphoma working group of the LYSA (Lymphoma Study Association). Except for two MEITL cases (MEITL 061 and 077), all patients had been included and described in earlier studies [20, 21]. Supplementary Table S1 briefly summarizes the clinicopathological features at initial diagnosis, while Supplementary Fig. S1 shows the number of samples included in each analysis and their overlaps. The median age at diagnosis was 64 (range: 34-86) years for EATLs and 67 (range: 29–91) years for MEITLs, with a male: female ratio of 2:1 and 1:1, respectively. Fourteen out of 30 (47%) EATLs had a confirmed diagnosis of celiac disease (CeD), while none of the MEITL patients had a history of CeD. The study protocol was approved by the local ethical committees: the Commission cantonale d’éthique de la recherche sur l’être humain (CER-VD, protocol 382/14), the Comité de Protection des Personnes-Ile-de-France IX (CPP08/009) in accordance with the Declaration of Helsinki. Demographic and clinical data were obtained from the patient’s files by the treating physicians or from the histopathological reports of the referring institutions.

### Immunohistochemistry

MMR protein (MLH1, MSH2, MSH6 and PMS2) immunohistochemistry (IHC) was performed on 4 µm formalin fixed paraffin embedded (FFPE) sections on automated immunostainers according to manufacturer recommendations (BenchMark XT and Ultra; Ventana Medical Systems, Tucson, AZ, USA), using the Ventana antibody panel validated for diagnostics: anti-MLH1 (M1) mouse monoclonal antibody, anti-PMS2 (A16-4) mouse monoclonal antibody, anti-MSH2 (G219-1129) mouse monoclonal antibody and anti-MSH6 (SP93) rabbit monoclonal antibody (Ventana, Tucson, AZ, USA). dMMR was defined as the complete loss of nuclear expression of any of the four MMR proteins, in the presence of positive staining of internal controls (reactive lymphocytes and/or stromal cells). Nuclear staining of the four MMR proteins in all tumor cells was considered proficient MMR (pMMR).

The staining was performed on sections from tissue microarray (TMA) blocks containing 21 EATLs and 53 MEITLs (two cores of 1 mm diameter per case). For the other cases (9 EATLs and 3 MEITLs) as well as for those cases showing dMMR in TMA slides, MMR protein immunostains were evaluated on whole tumor sections. In addition, partial expression of MMR proteins (presence of both positively and negatively stained nuclei) was also recorded.

For the evaluation of tumor infiltration lymphocytes (TILs), we counted intratumoral CD5+ cells by IHC in five representative fields captured at 40x magnification (area of 0.21 mm^2^) of immunostained whole tumor sections in a series of 32 MEITL cases. By this approach, we would capture the whole non-tumoral TCRαβ T-cell populations, as MEITL cases are almost constantly CD3+ and CD8 + , but CD5-. Staining was quantified using QuPath digital pathology open-source software (v0.5.0), as previously described [21]. Chromogenic slides were digitalized and evaluated as previously reported [20]. The antibodies used in this study are listed in Supplementary Table S2.

### Whole exome sequencing

Whole-exome sequencing (WES) data of 63 ITCL (26 EATLs, 37 MEITLs) and 60 matched normal extracted from distant non-tumoral FFPE tissues were previously generated and herein mined [21]. Variant calls and CNV analysis were performed as previously described [21]. TMB was computed by dividing the number of non-synonymous somatic mutations (missense, nonsense, frameshift, and in frame-indel) by the size of the exome panel covering the coding sequence of the human genome: specifically, 33.2 Mb for the IDT panel and 33.9 Mb for the Agilent v5 panel. TMB for other lymphoma entities and other cancers was obtained from the literature (see citations in Supplementary Table S3) or from TCGA (colorectal cancer (CRC), gastric cancer (GC), endometrial carcinoma (ENCA), ovarian cancer (OV), and renal cell carcinoma (RCC); source cBioportal - cBioPortal for Cancer Genomics).

MSI was assessed from WES data in 25 EATLs and 35 MEITLs for which matched normal was available using MANTIS (v1.04) [26] and MSIsenser-pro (v1.2.0) [27]. These algorithms measure the proportion of homopolymers and microsatellite sites in the covered part of the genome, showing a shift in their length when tumor and matched-normal sequences are compared. Single Base Substitution (SBS) signature analysis was performed using *musicatk* (v1.4.0) package in R (v4.1.2).

### Microsatellite instability assessment by PCR-based analysis

PCR analysis was performed in 46 cases (15 EATLs and 31 MEITLs), in duplicate and in parallel in tumor and matched normal samples, starting from 9 ng of genomic DNA, using 5 well-established mononucleotide repeat markers (BAT25, BAT26, NR21, NR24 and NR27) and the following conditions: 1X PCR buffer, MgCl2 2 mM, 0.75 to 2.5 μM of primers, dNTP 0.2 mM and 1U of Taq polymerase (Platinum Taq, Invirogen, Waltham, MA, USA). PCR was performed with a 5-minute hot-start at 95 °C, followed by 40 cycles with 30-sec denaturation at 95 °C, 45-second annealing at 55°C, and 30-second elongation at 72 °C, with a final 5-minute elongation at the end. The generated fragments were separated by capillary electrophoresis on the Applied Biosystems 3500 Series Genetic Analyzer (Thermo Fisher, Waltham, MA, USA) and analyzed by GeneMapper software (version4.1 - Thermo Fisher).

PCR results were interpreted as follows: MSS, none of the markers unstable; MSI-L, only one marker unstable, and MSI-H, ≥ 2 unstable markers. Since the clinical and biological significance of MSI-L remains controversial, we grouped MSI-L and MSS cases in one category “MSI-L/MSS” for analysis [1, 28].

### *MLH1* promoter DNA methylation status

*MLH1* promoter methylation status was assessed starting from the DNA methylation profiles generated using the Infinium MethylationEPIC BeadChip on 51 ITCLs (26 EATLs and 35 MEITLs) as previously described [21]. We analyzed the β values of four specific *MLH1* CpG sites (cg23658326, cg11600697, cg21490561, and cg00893636) that have been reported to predict the DNA methylation status of the *MLH1* gene promoter [29].

### Statistical methods

Categorical variables were compared using Fisher’s exact or chi-square test, while continuous variables were analyzed using two-sided nonparametric Mann–Whitney U test, if not otherwise specified. Multiple testing adjustments were performed using Holm-Bonferroni or Benjamini-Hochberg methods. Estimates of overall survival were constructed using the Kaplan-Meier method. Statistical analysis was carried out using R (v4.1.2).

## Results

### Primary ITCLs show a relatively high TMB

**Fig. 1:**
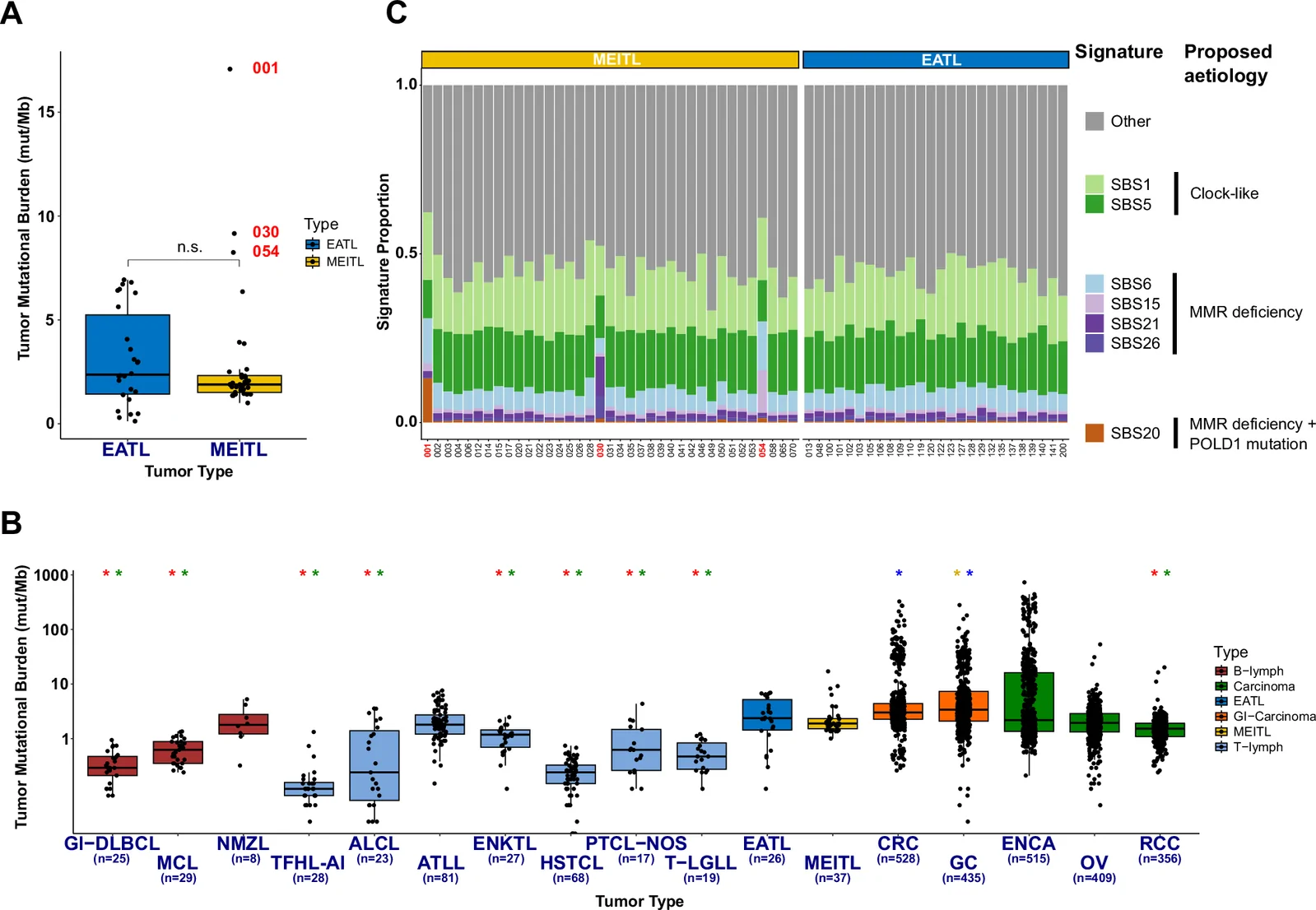
Tumor mutational burden (TMB) and mutational signatures. **A** Box and whiskers plots showing TMBs observed in 26 EATLs and 37 MEITLs. MEITL001, MEITL030 and MEITL054 cases showed the highest TMB values of the cohort. No statistically significant difference (n.s.) was observed between the two entities. **B** TMB distribution of the ITCLs (i.e., EATLs and MEITLs) is plotted against that of other T- and B-cell lymphomas and selected carcinomas with higher prevalence of MSI. The top, middle, and bottom lines of the boxes show the first, the second (median), and the third quartiles of the TMB values, respectively. Whiskers represent the values that are 1.5 times the interquartile range (IQR) above or below the first and the third quartiles. The number of samples per tumor type is reported under the boxplot. The presence of colored stars on the top of the boxplot indicates a significantly different mutational level compared to EATLs or MEITLs. Specifically, red or green stars indicate significantly lower; blue or yellow stars significantly higher TMB values than MEITLs or EATLs, respectively. Results are also summarized in Table 1. ITCLs showed higher TMB compared to several B- and T-cell lymphomas, such as TFHL-AI, ALCL, ENKTL, GI-DLBCL, HSTCL, MCL, PTCL-NOS, and T-LGL, and similar to carcinomas. **C** Contribution of single-base substitution (SBS) mutational signatures in 26 EATLs and 37 MEITLs. The most represented SBS signatures are reported in colors, while those with a lower contribution are merged and shown in grey. MEITL001, MEITL030, and MEITL054 (labeled in bold red) showed a distinctive pattern characterized by SBS associated with MMR deficiency (SBS6, SBS15, SBS20, SBS21, and SBS26). Other SBS signatures observed across all the patients were mainly clock-like (SBS1 and SBS5). Abbreviations: GI-DLBCL Gastrointestinal Diffuse large B-cell lymphoma, MCL Mantle cell lymphoma, NMZL Nodal marginal zone B cell lymphoma, TFHL-AI Nodal TFH cell lymphoma, angioimmunoblastic type, ALCL Anaplastic large-cell lymphoma, ATLL Adult T-cell leukemia/lymphoma, ENKTL Extranodal natural killer (NK)/T cell lymphoma, HSTCL Hepatosplenic T-cell Lymphoma, PTCL-NOS Peripheral T-cell lymphoma, not otherwise specified, T-LGLL T-cell large granular lymphocyte leukemia, EATL Enteropathy-associated T-cell lymphoma, MEITL Monomorphic epitheliotropic intestinal T-cell lymphoma, CRC Colorectal carcinoma, GC Gastric carcinoma, ENCA Endometrial carcinoma, OV Ovarian carcinoma, RCC Renal cell carcinoma.

As previously described [21], whole-exome sequencing (WES) analysis of 26 EATLs and 37 MEITLs identified a total of 7101 somatic mutations (5344 missense, 904 splice site, 473 frameshift, 290 nonsense, 72 in-frame-indels, 9 start lost, and 9 stop lost), with a median mutation count of 91 (range: 4-254) for EATLs and 72 (range: 41-839) for MEITLs. The overall median TMB was 2.4 mutations/Mb in EATLs (range: 0.1-6.9) and 1.9 mutations/Mb in MEITLs (range: 1-17.1) (*p* = 0.328, not significant). Notably, there were 3 MEITL tumors with much higher TMB, namely cases MEITL001, MEITL030, and MEITL054, with 17.1, 9.2, and 8.3 non-synonymous somatic mutations/Mb, respectively (Fig. 1A). Next, we compared TMB observed in our ITCL series versus those of other tumor entities obtained by collecting their mutation profiles from literature and TCGA database (full list of references in Supplementary Table S3). Noteworthy, most of the T- and B-cell lymphoma entities, including gastrointestinal diffuse large B-cell lymphoma (GI-DLBCL), mantle cell lymphoma (MCL), nodal TFH cell lymphoma, angioimmunoblastic type (TFHL-AI), anaplastic large-cell lymphoma (ALCL), extranodal natural killer (NK)/T cell lymphoma (ENKTL), hepatosplenic T-cell lymphoma (HSTCL), peripheral T-cell lymphoma, not otherwise specified (PTCL-NOS) and T-cell large granular lymphocyte leukemia (T-LGLL), showed TMBs ranging between 0.1 and 1.2 non-synonymous somatic mutations/Mb, which were significantly lower than the levels observed in ITCLs (*p* < 0.001). Moreover, ITCLs displayed TMBs comparable to those of ovarian and colorectal carcinomas, which are tumors known to be associated with MSI status (Fig. 1B and Table 1).

**Table 1:** Table summarizing comparisons of TMB levels between ITCLs and the other tumor entities using the Wilcoxon signed-rank test and adjusted *p*-values for multiple testing.

Type	*N*. patients	TMB (median - range)	EATL vs Other Types (adj. P. Values)	MEITL vs Other Types (adj. *P*. Values)
**EATL**	26	2.4 (0.1–6.9)	-	0.344
**MEITL**	37	1.9 (1–17.1)	0.344	-
**TFHL-AI**	28	0.1 (0–1.3)	**<0.001**	**<0.001**
**ALCL**	23	0.2 (0–3.6)	**<0.001**	**<0.001**
**ATLL**	81	1.8 (0.1–7.6)	0.156	0.323
**CRC**	528	3 (0.3–326)	0.102	**<0.001**
**ENCA**	515	2.2 (0.2–731.2)	0.181	0.222
**ENKTL**	27	1.2 (0.1–2.5)	**0.001**	**<0.001**
**GC**	435	3.4 (0–280.7)	**0.039**	**<0.001**
**GI-DLBCL**	25	0.3 (0.1–0.9)	**<0.001**	**<0.001**
**HS-TCL**	68	0.2 (0–0.7)	**<0.001**	**<0.001**
**MCL**	29	0.6 (0.2–1.4)	**<0.001**	**<0.001**
**NMZL**	8	1.8 (0.3–5.3)	0.42	0.53
**OV**	409	1.9 (0.1–53.3)	0.158	0.609
**PTCL-NOS**	17	0.6 (0.1–4.3)	**0.001**	**<0.001**
**RCC**	356	1.5 (0.2–20.2)	**0.001**	**<0.001**
**T-LGLL**	19	0.5 (0.1–1.2)	**<0.001**	**<0.001**

ITCLs showed higher TMB compared to several B and T lymphomas, such as nTFHL-AI, ALCL, ENKTL, GI-DLBCL, HSTCL, MCL, PTCL-NOS, and T-LGLL, and similar to carcinomas.

### A subgroup of MEITL is characterized by MSI-H status associated with dMMR mutational signatures and MLH1/PMS2 protein loss

**Fig. 2:**
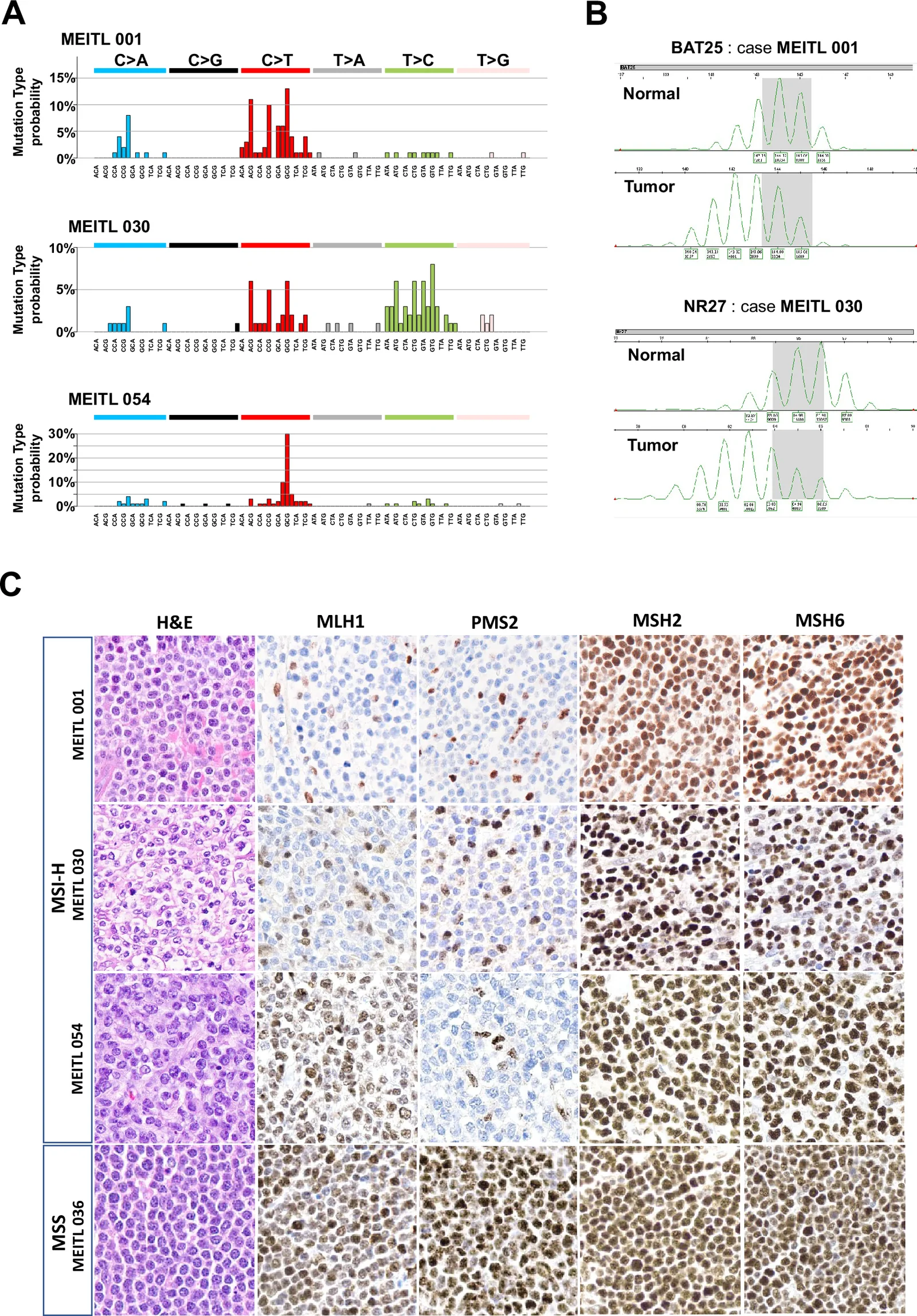
Mutational signatures, microsatellite markers, and MMR protein expression in three MSI-H MEITL tumors. **A** Distribution of 96 trinucleotide mutation spectrum for the MSI-H MEITL tumors. The height of each bar indicates the proportion of mutations of each trinucleotide class. The single-base mutations are indicated at the top of the plot along with the color code. **B** Example of PCR-based MSI analysis on two patients. Electropherograms of BAT25 and NR27 microsatellite markers on MEITL001 and MEITL030 (both MSI-H), compared with the DNA from matched healthy cells (control; MS-stable). Both markers showed a typical left shift of 2/3pb in their length in the tumor component, indicative of microsatellite instability. **C** MMR protein expression in the three MSI-H MEITLs and one MSS MEITL. MEITL001 and MEITL030 showed a complete loss of expression of MLH1 and PMS2, while MEITL054 was characterized by an isolated and complete loss of PMS2, with preserved MLH1 expression. MEITL036 (a MSS case) showed preserved expression of all four MMR proteins (H&E and immunohistochemistry, all 400x).

Using WES data of 60 ITCLs with available tumor and matched normal, we assessed MSI status by two approaches: analyzing the proportion of unstable microsatellite sites with MANTIS and MSISensor-pro algorithms or assessing single-base substitution (SBS) mutation signatures. With a former approach, only a single case, MEITL001, exhibited a score above the threshold that defines MSI-H status (Supplementary Table S4). It should be noted that those thresholds were defined using solid tumors in which MSI-H status represents a major tumor driver in their tumorigenesis. Moreover, by mutation signature assessment, the three MEITLs with the highest TMB (cases MEITL001, MEITL030, and MEITL054) showed a greater contribution from signatures associated with dMMR, specifically SBS6, SBS15, SBS20, SBS21, and SBS26 (Figs. 1C, 2A). To validate these findings, we performed a PCR-based MSI assay targeting five well-established mononucleotide repeat markers in 46 samples with matched normal DNA (15 EATLs, 31 MEITLs) [30] (Supplementary Table S5). Consistent with the above findings, only the same three MEITLs (MEITL001, MEITL030, and MEITL054) showed MSI-H status with shifts in multiple markers compared to their matched normal DNA (Fig. 2B). Notably, the size of the peak shifts ranged only between 1 and 3pb, which is at the lower end of the distribution observed in other MSI-H tumors such as colorectal cancers [31, 32]. On the other side, one out of 15 EATLs (EATL109) was classified as MSI-L, showing only one shifting marker, while all other MEITL and EATL cases were MSS.

IHC for MLH1, PMS2, MSH2, and MSH6 was performed in 74 cases (21 EATL, 53 MEITL). The three MSI-H MEITL cases showed dMMR. Specifically, MEITL001 and MEITL030 showed a complete loss of expression of MLH1 and PMS2, and MEITL054 showed a complete loss of PMS2 (Fig. 2C). All EATLs and the other 50 MEITLs had preserved expression of the four proteins (pMMR). Of note, three MEITL cases (MEITL024, MEITL058, and MEITL061) showed expression of MLH1 protein in a fraction of the tumor cells (20, 24, and 18% positive nuclei, respectively). These samples did not show an increased TMB or a mutational signature associated with dMMR, suggesting that partial expression of MMR proteins was not sufficient to induce an MSI-H phenotype. Moreover, MEITL058 was tested by PCR and was confirmed microsatellite stable (MSS) (Supplementary fig. S2).

### dMMR is due to *MLH1* and *PMS2* locus biallelic deletion

To investigate the molecular mechanisms underlying the MSI-H phenotype associated with loss of MLH1 and/or PMS2 protein expression, we analyzed the WES data for mutations and CNVs in key MMR genes, namely *MLH1*, *PMS2*, *MSH2*, and *MSH6*.

No germline pathogenetic variants or somatic small mutations were detected in these genes across the 63 samples with available WES data. Additionally, DNA methylation profiling of 61 samples (26 EATLs and 35 MEITLs) revealed no evidence of *MLH1* promoter hypermethylation.

**Fig. 3:**
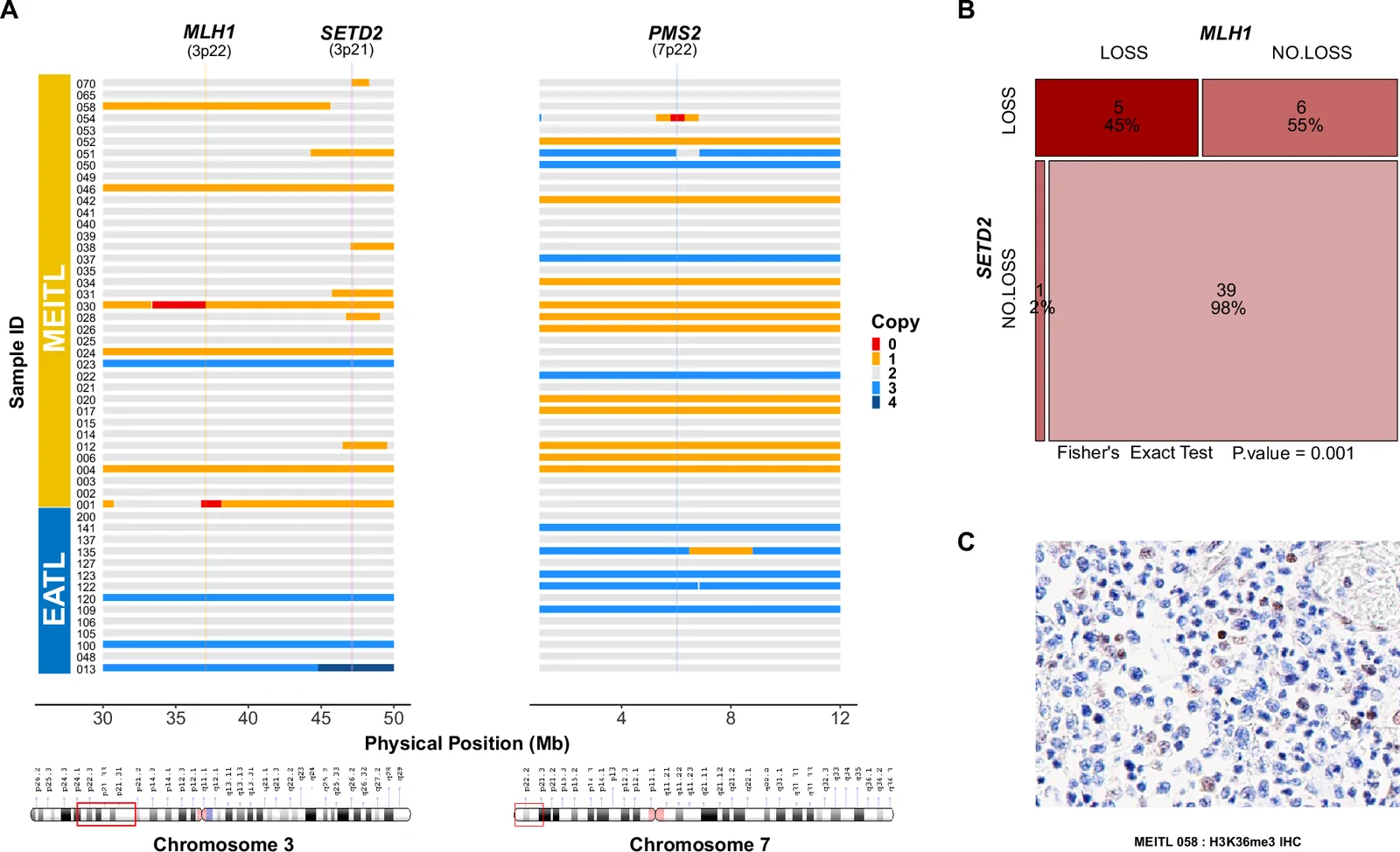
Association of *MLH1* deletions with *SETD2* alterations. **A** Copy number variations (CNV) observed within the 3p arm (between 30 and 50 Mb region) and 7p arm (between 0.2 and 12 Mb region) across 37 MEITLs and 14 EATLs. Region 3p showed frequent deletions (12/37) in MEITL, almost always including *SETD2* gene locus. Six of those encompassed also the *MLH1* gene locus, with two (MEITL001 and MEITL030) presenting a biallelic loss of the gene. EATLs showed no deletion but copy number gains in 3 cases. The region in 7p was also exclusively lost in MEITLs (12/37), with patient MEITL054 showing biallelic loss of the *PMS2* locus. Gain of one copy encompassing *PMS2* locus was detected in both EATLs (5/14) and MEITLs (3/37). Regions with diploid copy numbers are shown in gray, one copy deletion in orange, biallelic deletion in red, one copy gain in blue, and 2 copies gain in dark blue. **B** Mosaic plot of 51 ITCLs showing that deletions of *MLH1* locus cooccurred with those of *SETD2* in a significant manner (Fisher's exact test). The width of the columns and rows are proportional to the number of samples with or without loss of the loci for *MLH1* and *SETD2*, respectively. The annotations report on the numbers and proportions of samples in each group. **C** H3K36me3 immunostaining in case MEITL058, which showed one mutation of *SETD2* and *MLH1* monoallelic deletion without *SETD2* deletion; lack of H3K36me3 reflects altered SETD2 function (immunohistochemistry, 400x).

Moreover, CNV analysis was performed on 51 samples (37 MEITLs and 14 EATLs) with sufficient tumor content and sequencing coverage. Chromosomal deletions encompassing the whole *MLH1* (3p22) locus were identified in 6 of 37 MEITL cases (16%), including four cases with monoallelic deletion and two cases with biallelic deletions. Another MEITL case (MEITL023) exhibited a single-copy gain (Fig. 3A). The median deletion size in chromosome 3p encompassing the *MLH1* gene was 36.9 Mb (range: 1.4–118.1 Mb), with two focal biallelic deletions having a minimal common deleted region between the two alleles measuring 1.4 and 3.7 Mb. Notably, these biallelic deletions corresponded to the two MSI-H MEITLs (MEITL001 and MEITL030) with complete loss of MLH1 and PMS2 protein expression. Interestingly, two of the three cases with partial MLH1 protein expression (MEITL024 and MEITL058) harbored monoallelic *MLH1* deletions. In EATLs, no *MLH1* deletions were observed, and three cases (21%) showed single-copy gains (median size: 87.8 Mb; range: 44.6–93.2 Mb).

*PMS2* (7p22) deletions were detected in 12 of 37 MEITL cases (32%), including eleven monoallelic losses (all pMMR) and one biallelic deletion in MEITL054 (MSI-H and PMS2 protein loss). The median deletion size was 57.3 Mb (range: 0.5–62.7 Mb), with the focal biallelic deletion encompassing *PMS2* spanning only 510 kb. Three MEITLs exhibited single-copy gains involving full chromosome 7 (size: 158.7 Mb). No *PMS2* deletions were observed in EATLs, but five cases (36%) showed single-copy gains (median size: 37.2 Mb; range: 6.3–158.8 Mb). CNV analysis of methylation array data corroborated the complete loss of *MLH1* and *PMS2* loci identified by WES (data not shown).

Finally, one MEITL case (MEITL023) showed monoallelic loss of both *MSH2* and *MSH6*, with inconclusive MMR immunostaining results.

### *MLH1* and *SETD2* locus deletions co-occur in MEITL

Given the relatively close genomic proximity ( ~ 10 Mb) between *MLH1* (3p22) and *SETD2* (3p21), the latter being altered in over 95% of MEITL cases through mutation and/or deletion [20], we investigated whether their deletions co-occurred. Notably, in 5 of 6 MEITLs, monoallelic or biallelic *MLH1* deletions also affected the *SETD2* locus. Six other MEITLs had heterozygous chromosome 3p-deleted segments encompassing *SETD2* but not *MLH1* (Fig. 3A). Overall, there was a significant association between *MLH1* and *SETD2* deletions (Fisher-test *p* = 0.001), supporting the hypothesis that *MLH1* first allele loss may occur as a ‘bystander’ event due to broader chromosomal alterations targeting *SETD2* (Fig. 3B). Interestingly, in the single case (MEITL058) where a deletion affected *MLH1* but not *SETD*2, only one *SETD2* mutation was present. This deletion was only approximately 1 Mb upstream of the *SETD2* locus. This tumor also showed loss of SETD2 protein expression and absence of H3K36me3 staining by IHC, suggesting that the adjacent upstream deletion could represent the second hit, contributing to impaired SETD2 activity (Fig. 3B, C).

### Morphologic and clinical features of MSI-H MEITL cases

**Fig. 4:**
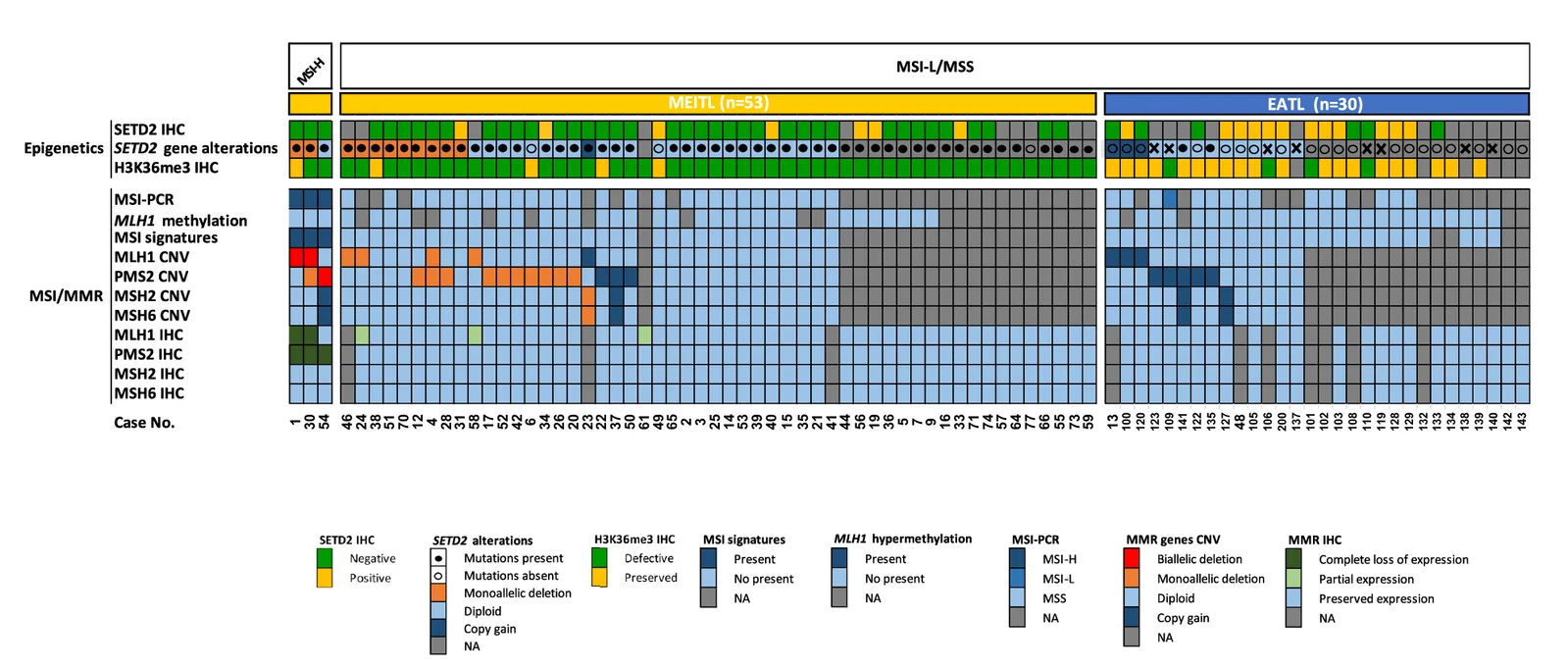
Heatmap of molecular alterations associated with MMR status and SETD2 in the cohort. Heatmap representation of the molecular features, including *SETD2/*H3K36me3 axis and mismatch repair deficiency (dMMR)/Microsatellite instability (MSI) status in 30 EATLs and 56 MEITLs. Patients are displayed as columns. The three MEITL cases with MSI-high are shown on the left. Abbreviations: CNV copy number variation, IHC immunohistochemistry, MMR mismatch repair, MSI microsatellite instability, MSI-H microsatellite instability-high, MSI-L microsatellite instability-low, NA not available.

We next reviewed the clinical and pathological characteristics of the three MSI-H MEITL. MEITL001 and MEITL054 were males (aged 72 and 54, respectively) and MEITL030 was a female (aged 47). The three tumors developed in the small intestine, and they were cytologically classic with evident epitheliotropism and cytotoxic immunophenotype. MEITL001 and MEITL054 showed a typical MEITL immunophenotype with CD8 and CD56 positivity and TCRγδ isoform expression, with the latter case also presenting atypical morphological features, including necrosis and angiotropism. MEITL030 was CD8-negative, showed a weak and partial expression of CD56, and lacked expression of TCRγδ and αβ isoforms (TCR-silent). Concerning the classic molecular alterations observed in MEITLs, MEITL054 and MEITL030 carried nonsense mutations for the *SETD2* gene and associated loss of H3K36me3 trimethylation (Fig. 4). Besides, they showed missense mutations in *STAT5B*, with MEITL030 harboring also a *JAK3* mutation. MEITL001 did not carry *SETD2*, *STAT5B* or *JAK3* mutations, but a *TP53* mutation associated with protein over-expression [20]. (Supplementary Fig. S3A). Besides, MEITL001 tumor also exhibited a *POLE* mutation (c.5516 G > T; p.Arg1839Leu) located outside the canonical exonuclease domain, but lacked the characteristic mutational signature of POLE-driven hypermutation, supporting the interpretation that this was a passenger mutation.

Finally, we compared the density of intratumoral CD5+ TILs in MSI-H and MSS MEITL cases. Overall, we observed variable numbers of intratumoral CD5+ TILs (median: 44 CD5+ TILs/HPF; range: 6–359 CD5+ TILs/HPF), which did not differ according to the MSI status (median value: 11.6 in the three MSI-H cases vs 26.4 in 29 MSS MEITL cases, *p* = 0.26), Supplementary Fig. S3B). In addition, cellular deconvolution analysis performed from gene expression data using the CIBERSORT analytical tool (as described in [21], data not shown), showed no differences in CD4 memory activated, CD4 memory resting, CD4 Treg, NK activated, and NK resting signatures in the three MSI-H MEITLs compared to MSS cases.

The three patients with MSI-H MEITL underwent surgical tumor resection followed by CHOP-based chemotherapy. None of them received hematopoietic cell transplantation. Of note, MEITL030 presented a relapse five years after diagnosis. The three patients died from progressive disease at 17, 71, and 5 months, respectively, with a median overall survival (OS) of 17 months from initial diagnosis.

## Discussion

In this study, we explored the MSI status and dMMR mechanisms in a set of 86 ITCLs (30 EATLs, 56 MEITLs). Our findings revealed that, in our cohort, 6% of MEITL patients exhibit dMMR through genomic loss of *MLH1* or *PMS2* loci. Given that dMMR and MSI are associated with responses to immune checkpoint inhibitors in other cancers [33], this finding provides a biological rationale for exploring the potential efficacy of immune checkpoint inhibitors in this particularly unfavorable subtype of ITCL.

Overall, ITCLs, regardless of MMR status, showed higher TMB compared to other B- and T-cell lymphomas, and similar to those of some carcinomas [34]. Although the molecular mechanisms underlying this feature in ITCLs remain unknown, elevated TMB has been linked to alterations in epigenetic regulators such as *SETD2*, *ARID1A*, *KMT2C*, and *EP300* in MSS colorectal cancer [35]. In particular, *SETD2* mutations have been associated with significantly higher TMB in several solid tumors, including colorectal carcinoma, non-small cell lung cancer, melanoma, glioma, and pancreatic carcinoma [36]. Concerning dMMR/MSI-H, prevalence varies across cancers, with the highest observed in carcinomas, such as endometrial (ranging from 22-29%), ovarian (6-18%), colorectal (5–16%), and gastric (2–17%), depending also on the detection methods [37]. Studies in lymphoid neoplasms often involve small cohorts using variable methods of detection, making MSI frequency estimations not always comparable or reliable. In this regard, a few studies reported the presence of MSI-L cases in gastric MALT lymphoma (18%) [16], DLBCL (10%) [12], primary cutaneous lymphoma (3%) [17], and primary mediastinal large B-cell lymphoma (1%) [18]. In contrast, MSI-H cases appear less frequent and have been reported mainly in immunodeficiency-associated lymphomas, including HIV and post-transplant lymphomas (8% and 2% respectively) [19], as well as in DLBCL not associated with immunodeficiency (3%) [12] and in DLBCL transformed from gastric MALT lymphoma (4%) [16]. However, more recent studies have not confirmed the presence of MSI in HIV-associated DLBCL [38], gastric DLBCL and gastric MALT lymphoma [39] or Richter transformation-diffuse large B-cell lymphoma variant (RT-DLBCL) [40].

In ITCLs, Baumgärtner et al. explored the genomic instability of 26 cases (10 classified morphologically as “monomorphic”, 13 as “pleomorphic” and 3 as “unclassified”), using a battery of 47 microsatellite markers. Although they reported instability of at least one marker in 69% of cases (all MSI-L), the significance of such observation outside the canonical microsatellite regions remains elusive [15]. In the present study, three MEITL cases stood out from the rest of the cohort with notably higher TMB and dMMR-associated tumor signatures. Their MSI-H status was confirmed by PCR and IHC analysis, leading to a prevalence of MSI-H among MEITLs estimated at around 6% (3/53 by IHC cohort) and 0% in EATLs.

Analyses of WES and DNA methylation demonstrated that the observed dMMR phenotype is not due to germline pathogenic variants or small somatic mutations of the MMR genes, nor to *MLH1* promoter hypermethylation. These genetic alterations are rarely observed in lymphomas, where mechanisms driving dMMR in MSI-H are largely unknown [12, 16–19]. Instead, CNV analysis of these dMMR MEITL cases revealed that the culprit of the MMR protein expression loss was the biallelic deletions of the *MLH1* locus (3p22) in MEITL001 and MEITL030, or of the *PMS2* locus (7p22) in MEIT054. Interestingly, in a subset of MSS MEITL cases, monoallelic deletion of the *MLH1* locus, but not of the *PMS2* locus, was associated with partial MLH1 protein expression, suggesting a potential role of CNV in regulating *MLH1* expression levels without affecting TMB levels. Both 3p and 7p chromosome arm deletions have been previously reported in ITCLs, with frequencies corroborating our observations. In a series of 30 ITCLs analyzed by whole genome sequencing, Deleew et al. observed 3p22 locus deletions (2.3 Mb in length) in 6/30 (20%) of cases, which were more frequently seen in MEITLs (5/15, 33%) than in EATLs (1/15, 7%) [41]. But the MSI status was not investigated. Besides, they detected deletion of 7p21.3 with the same frequency and distribution of 3p22, although not always co-occurring in the same tumor.

We have previously found *SETD2* locus deletions in 16% of MEITL cases evaluated by FISH [20]. In the present study the presence of *MLH1* locus deletions was almost always associated with *SETD2* locus deletions, with a significant co-occurrence. Codeletion of *MLH1* and *SETD2* in 3p region has also been reported in clear cell renal cell carcinomas (ccRCC), where *SETD2* is one of the most frequently mutated genes ( ~ 13%) together with *VHL*, *PBRM1*, and *BAP1*, all located at chromosome 3p [42]. Thus, the frequent loss of 3p arm indicates a vulnerability of this region in both ccRCC and MEITL, which seem to rely on the functional loss of genes mapped to 3p for their tumorigenesis [43]. In that sense, *SETD2* acts as a tumor suppressor, being involved in various important cellular processes including transcriptional regulation, alternative RNA splicing, DNA damage repair, apoptotic response, interferon response, and genomic stability [44]. Furthermore, recent evidence indicates that SETD2 protects against genomic instability through its catalytic activity and H3K36 trimethylation by the maintenance of homologous recombination repair (HRR) and MMR in tumor cells, as well as by catalysis-independent function stabilizing the nuclear lamina [36, 45]. Markedly, the loss of one allele of the *SETD2* gene seems sufficient to induce defects in nuclear morphology and genome stability [45]. Therefore, these observations would support a scenario in which an initial hit on the *SETD2* gene, either by a loss-of-function mutation or deletion, could affect overall genome stability, increasing the risk of further chromosomal alterations. This is consistent with high chromosomal instability scores observed in MEITL [21]. We can speculate that this increased genomic instability and the fact that the *MLH1* locus might also be affected by the frequently observed deletion of *SETD2* at its proximity, raises the chance of biallelic deletions of the *MLH1* gene in the MEITL subtype. Additional evidence suggests that dMMR is unlikely to represent a primary driver in MSI-H MEITL cases but rather a late-occurring event. This is supported by the modest shifts observed in MSI peak markers, the low MANTIS and MSIsensor-pro scores, and the relatively low TMB compared to sporadic and hereditary MSI-H tumors [31, 32]. In fact, previous studies have demonstrated that these parameters are interrelated, with the magnitude of peak shifts positively correlating with the number of coding microsatellite mutations. The authors proposed that peak shift size reflects an accumulative process, proportional to the duration of tumor cell exposure to a defective MMR protein complex.

Moreover, growing evidence indicates that the quantity, distribution, and composition of TILs within the tumor microenvironment are associated with favorable responses to ICIs in solid tumors. High densities of intratumoral CD3⁺, CD4⁺, CD8⁺, and CD20⁺ TILs are typically linked to antitumor immune activity, whereas FOXP3⁺ regulatory T cells are often associated with immunosuppression and poorer prognosis [46]. Although an increased number of TILs is usually associated with MSI-H status, in early-stage colorectal cancer, up to 40% of MSI-H cases exhibit low TIL counts, while approximately 50% of MSS cases show high TIL infiltration [47]. We found no difference in the levels of intratumoral TIL counts between MSI-H and MSS MEITLs, a finding corroborated by deconvolution analysis based on gene expression profiling (data not shown).

Finally, the discovery of MSI in a subset of MEITLs may be clinically relevant since MSI status is nowadays regarded as an indicative biomarker of patients’ response to therapy and survival. In colorectal carcinoma, MSI-H tumors generally have better outcomes than MSS tumors, partly due to enhanced lymphocytic responses against neoantigens generated by dMMR [47]. In lymphomas, particularly in DLBCL, MSI-L status was correlated with poor response to R-CHOP/CHOP chemotherapy, and multivariate analysis showed that MSI-L was an independent predictive factor for non-CR to R-CHOP/CHOP chemotherapy, with a trend of MSI-H with favorable PFS and OS [12]. The restricted number of MSI cases in our study limits statistical analysis of clinico-pathological variables and survival.

Overall, our study demonstrates that MEITL occasionally exhibits dMMR due to genomic loss of the *MLH1* or *PMS2* loci. It seems that these events occur during tumor progression, rather than being an initial tumorigenic driver. These findings provide a biological rationale for dMMR screening and for investigating the impact of immunotherapy, though further studies are needed to clarify the prognostic and predictive value of MSI in MEITL.

## Supplementary information

Supplementary figure and table legends

Supplementary figureS1

Supplementary figure S2

Supplementary figure S3

Supplementary Table S1

Supplementary Table S2

Supplementary Table S3

Supplementary Table S4

Supplementary Table S5
