# Supplementary material for: Genomic loss of MLH1/PMS2 loci defines a mismatch repair deficient subgroup in monomorphic epitheliotropic intestinal T-cell lymphoma
Source: Blood Cancer J. 2026 Jul 3;16(1):161. doi: 10.1038/s41408-026-01562-3 (PMC13601611; doi:10.1038/s41408-026-01562-3)

Supplementary figure S2

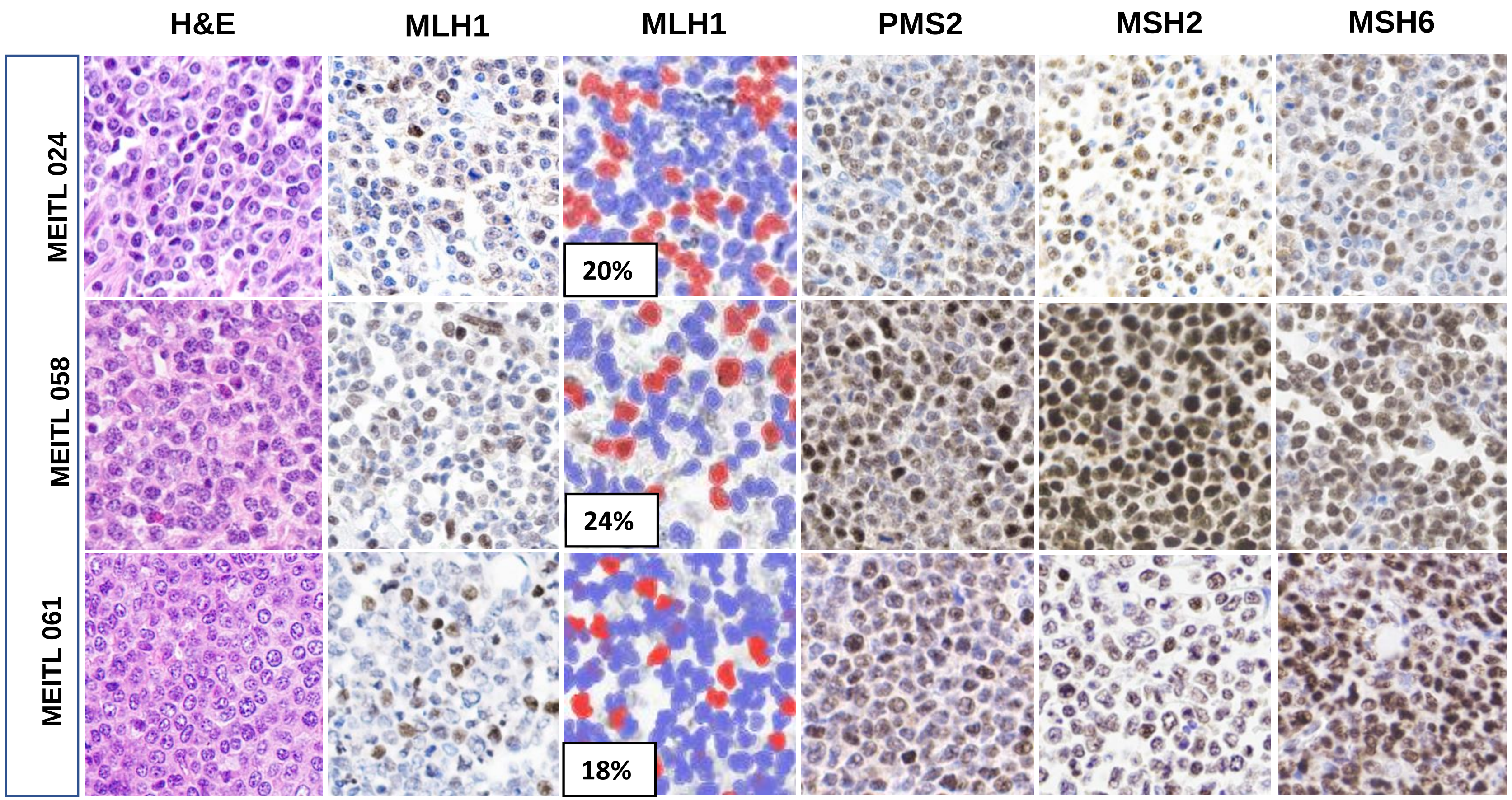

Supplement: Supplementary file 2 [file 41408_2026_1562_MOESM3_ESM.pdf]
