# Supplementary material for: Genomic loss of MLH1/PMS2 loci defines a mismatch repair deficient subgroup in monomorphic epitheliotropic intestinal T-cell lymphoma
Source: Blood Cancer J. 2026 Jul 3;16(1):161. doi: 10.1038/s41408-026-01562-3 (PMC13601611; doi:10.1038/s41408-026-01562-3)
Supplement: Supplementary file 4 [file 41408_2026_1562_MOESM1_ESM.docx]

**Supplementary figure and supplementary table legends**

**Supplementary Figure S1:** Venn Diagrams showing the number of cases included in this study according to assays and survival data availability.

**Supplementary Figure S2**: Composite image displaying the three MEITL cases (MEITL024, 058 and 061) with partial expression of MLH1, without loss of expression of PMS2, MSH2 or MSH6 (H&E and immunohistochemistry, all 400x). For MLH1 immunohistochemical evaluation, representative images of the quantifications of MLH1 expressing tumor cells (20, 24, and 18% respectively) analyzed by QuPath (version 0.5.0) are shown in the third column.

**Supplementary Figure S3**: **A**) Morphology and immunophenotype of MSI-H MEITLs. MEITL001 and MEITL054 show the typical MEITL immunophenotype (CD8+ and CD56+), while tumor cells in MEITL030 had a weak expression of CD56 and were negative for CD8 (H&E and immunohistochemistry, all 400x). **B**) No statistically significant (n.s) association was observed between MSI status and the number of intratumoral CD5+ TILs in the MEITL cohort (N=32: 3 MSI-H and 29 MSS).

**Supplementary Table S1**. Clinicopathological features of MEITL and EATL

**Supplementary Table S2**. List of antibodies used for immunohistochemical studies.

**Supplementary Table S3**. Number of non-synonimous mutations and TMB from other lymphoma entities obtained from literature or from TCGA database (Source).

**Supplementary Table S4**. MSI score and status assessed from WES data using MANTIS and MSIsenser-pro algorithms.

**Supplementary Table S5**. Results of the PCR and fragment analysis of the 5 mononucleotide repeats regions (BAT25, BAT26, NR21, NR24 and NR27).
