# Supplementary material for: Genomic loss of MLH1/PMS2 loci defines a mismatch repair deficient subgroup in monomorphic epitheliotropic intestinal T-cell lymphoma
Source: Blood Cancer J. 2026 Jul 3;16(1):161. doi: 10.1038/s41408-026-01562-3 (PMC13601611; doi:10.1038/s41408-026-01562-3)
Supplement: Supplementary file 5 [file 41408_2026_1562_MOESM5_ESM.docx]

**Supplementary Table S1. Clinicopathological features of MEITL and EATL**

|  | | **MEITL**  **(n=56)** | **EATL**  **(n=30)** | **Adjusted p value*** |
| --- | --- | --- | --- | --- |
| **Clinical features** | | | | |
| Reported coeliac disease | | 0/56 (0%) | 14/30 (47%) | **<0.001** |
| Age, median, years (range, median) | | 67 (29-91) | 64 (34-86) | 1.000 |
| Gender (male/female) | | 28:28 | 19:11 | 1.000 |
| Overall survival (months) 95% CI | | 5.8 (96-257) | 7.9 (0-187) | 1.000 |
| **Morphological features** | | | | |
| Cytology | |  |  |  |
| Monomorphic | | 42/56 (75%) | NA |  |
| Non-monomorphic | | 14/56 (25%) | NA |  |
| Immunoblastic | | NA | 15/30 (50%) |  |
| Anaplastic | | 1/56 (2%) | 15/30 (50%) | **<0.001** |
| Necrosis | | 10/56 (18%) | 22/30 (73%) | **<0.001** |
| Starry-sky | | 7/56 (12%) | 3/30 (10%) | 1.000 |
| Angiotropism | | 15/56 (27%) | 19/29 (65%) | **0.024** |
| **Immunological markers** | | | | |
|  | CD8 | 51/56 (91%) | 4/30 (13%) | **<0.001** |
|  | CD56 | 50/56 (89%) | 2/28 (7%) | **<0.001** |
|  | CD3 | 56/56 (100%) | 27/30 (90%) | 1.000 |
|  | CD2 | 25/52 (48%) | 12/24 (50%) | 1.000 |
|  | CD5 | 2/56 (4%) | 2/30 (7%) | 1.000 |
|  | CD7 | 52/53 (98%) | 22/23 (95%) | 1.000 |
|  | CD4 | 3/56 (5%) | 2/27 (7%) | 1.000 |
|  | CD103 | 44/54 (81%) | 13/25 (52%) | 0.258 |
|  | CD30 | 1/49 (2%) | 25/29 (86%) | **<0.001** |
|  | TIA1 | 50/54 (92%) | 20/25 (80%) | 1.000 |
|  | Granzyme B | 42/54 (78%) | 19/28 (67%) | 1.000 |
|  | Perforin | 32/52 (61%) | 20/22 (90%) | 0.417 |
|  | CD20 | 10/54 (18%) | 1/29 (3%) | 1.000 |
|  | CD79a | 3/42 (7%) | 0/12 (0%) | 1.000 |
| **TCR expression** | | | | |
|  | TCRβ | 16/54 (29%) | 8/28 (29%) | 1.000 |
|  | TCRγδ | 28/55 (51%) | 8/27 (30%) | 1.000 |
|  | TCRαβ-TCRγδ+ | 22/53 (43%) | 5/27 (18%) | 1.000 |
|  | TCRαβ+TCRγδ- | 12/53 (22%) | 4/27 (15%) |  |
|  | TCRαβ+TCRγδ+ | 4/53 (7%) | 3/27 (10%) |  |
|  | TCRαβ-TCRγδ- | 15/53 (28%) | 15/27 (55%) |  |
| **EBER-ISH** | | 0/56 (0%) | 0/30 (0%) | NA |

*p values adjusted using Holm-Bonferroni method
