# Supplementary material for: Genomic loss of MLH1/PMS2 loci defines a mismatch repair deficient subgroup in monomorphic epitheliotropic intestinal T-cell lymphoma
Source: Blood Cancer J. 2026 Jul 3;16(1):161. doi: 10.1038/s41408-026-01562-3 (PMC13601611; doi:10.1038/s41408-026-01562-3)
Supplement: Supplementary file 6 [file 41408_2026_1562_MOESM6_ESM.docx]

**Supplementary Table S2.** List of antibodies used for immunohistochemical studies.

| **Antibody** | **Clone** | **Source** | **Dilution** |
| --- | --- | --- | --- |
| MLH1 | M1 | Ventana Medical Systems, Tucson, AZ | RTU |
| MSH2 | G219-1129 | Ventana Medical Systems, Tucson, AZ | RTU |
| MSH6 | SP93 | Ventana Medical Systems, Tucson, AZ | RTU |
| PMS2 | A16-4 | Ventana Medical Systems, Tucson, AZ | RTU |
| CD3 | 2GV6 | Ventana Medical Systems, Tucson, AZ | RTU |
| CD20 | L26 | Novocastra, Newcastle, UK | 1/400 |
| CD2 | AB75 | Novocastra, Newcastle, UK | 1/30 |
| CD4 | SP35 | Ventana Medical Systems, Tucson, AZ | RTU |
| CD5 | SP19 | Abcam, Cambridge, UK | 1/40 |
| CD7 | CBC.37 | DakoCytomation; Agilent Technologies, Santa Clara, CA | 1/25 |
| CD8 | C8/144B | DakoCytomation; Agilent Technologies, Santa Clara, CA | 1/30 |
| CD30 | Ber-H2 | DakoCytomation; Agilent Technologies, Santa Clara, CA | 1/30 |
| CD56 | CD564 | Novocastra, Newcastle, UK | 1/25 |
| CD79a | JCB117 | DakoCytomation; Agilent Technologies, Santa Clara, CA | 1/50 |
| CD103 | EPR4166 | GeneTex, Irvine, CA | 1/300 |
| TIA-1 | 2G9A10F5 | Beckman Coulter, Brea, CA | 1/1000 |
| Granzyme B | GrB-7 | Monosan, Uden, The Netherlands | 1/30 |
| Perforin | 5B10 | Diagnostic Biosystem, Pleasanton, CA | 1/10 |
| Ki-67 | MIB-1 | DakoCytomation, Agilent Technologies, Santa Clara, CA | 1/50 |
| TCRb-F1 | 8A3 | Thermo Fisher Scientific, Waltham, MA | 1/30 |
| TCRγ | 3.20 | Thermo Fisher Scientific, Waltham, MA | 1/100 |
| TCRδ | H41 | Santa Cruz, Dallas, TX | 1/200 |
| SETD2 | Polyclonal | Sigma, Sigma-Aldrich, St. Louis, MO | 1/200 |
| H3K36me3 | Polyclonal | Abcam, Cambridge, UK | 1/200 |
| H3K36me2 | Polyclonal | Abcam, Cambridge, UK | 1/250 |
| MYC | Y69 | Ventana Medical Systems, Tucson, AZ | RTU |
| P53 | DO-7 | DakoCytomation, Agilent Technologies, Santa Clara, CA | 1/500 |

*Abbreviation: RTU, ready-to-use*
